# The role of social networks in supporting self-management in CKD: a narrative synthesis

**DOI:** 10.1186/s12882-025-04658-3

**Published:** 2025-12-09

**Authors:** Becky Bonfield, Kristin Veighey, Tom Blakeman, Emma Murphy, Ivaylo Ivanov Vassilev

**Affiliations:** 1 School of Health Sciences, University of Southampton, University Road, Southampton, UK; 2 University Hospital Southampton NHS Foundation Trust, Southampton, UK; 3 Primary Care Research Centre, University of Southampton, University Road, Southampton, UK; 4 Division of Population Health, Health Services Research and Primary Care, School of Health Sciences, The University of Manchester, Manchester, UK; 5 NIHR Greater Manchester Patient Safety Research Collaborative, The University of Manchester, Manchester, UK; 6 Centre for Care Excellence, Coventry University and University Hospitals Coventry and Warwickshire NHS Trust, Coventry, UK; 7 Institute for Cardio-Metabolic Medicine, University Hospitals Coventry and Warwickshire NHS Trust, Coventry, UK

**Keywords:** Chronic kidney disease, Social networks, Self-management, Peer support

## Abstract

**Background:**

Chronic kidney disease (CKD) is a growing global health concern requiring effective self-management to mitigate progression and improve quality of life. While self-management is increasingly recognised as a socially embedded practice, the specific contributions of social network members to this process in CKD are not well understood.

**Objective:**

To synthesise and interpret existing literature on how social networks support individuals living with early-stage CKD and identify gaps in understanding.

**Methods:**

We conducted a narrative review. Fourteen studies involving 560 participants—including individuals living with CKD, their social network members, healthcare professionals, peer mentors, and religious leaders—were analysed to explore the role of social networks in CKD self-management.

**Results:**

Four key themes emerged: (1) *The burden of kidney disease uncertainty* (2), *Everyday challenges of managing CKD* (3), *The loneliest disease*, and (4) *The role of peer support*. Participants frequently reported emotional distress linked to diagnostic uncertainty and inconsistent information, especially from non-specialist providers. CKD management posed significant practical and psychological burdens, particularly for caregivers, who often felt unsupported and invisible within healthcare systems. Peer support—both informal and formal—played a crucial role in reducing isolation and providing context-specific guidance, especially when tailored to individual preferences and illness trajectories.

**Conclusions:**

This is the first review to explore the role of social networks in supporting the self-management of people living with CKD. The studies highlighted that early-stage CKD is shaped by diagnostic uncertainty, limited formal support, and social invisibility. People with CKD rely on diverse social networks for self-management, yet unclear care pathways and inconsistent communication hinder this support. Integrated models that combine early specialist input, peer support, and trained generalists are needed. Recognising and supporting these networks is essential to reduce isolation, improve understanding, and enable meaningful engagement with self-management across the CKD trajectory.

## Background

 Chronic Kidney Disease (CKD) is defined as a reduction in kidney function for more than 90 days, and is classified based on cause, glomerular filtration rate (GFR) category and albuminuria category [1]. It ranges from early stages, often symptomatic, to Stage 5 kidney failure that may require dialysis or kidney transplant [2–5]. CKD is a global health issue that affects approximately 9% of the world’s population [6], with an estimated prevalence of 13% within the United Kingdom (UK) [7], which is continuing to rise Healthcare resource utilisation for people with CKD and its associated costs impose a significant burden on the healthcare system(accounting for 1.3% of NHS spending) [8], especially for those in advanced stages of CKD [9]. Alongside these financial and resource implications, CKD can have a detrimental effect on the physical and emotional well-being of people living with the disease, leading to a reduction in quality of life and psychological distress [3–5].

Early stages of CKD (Stages 1–3) may be asymptomatic, highlighting the importance of early detection and management to slow or halt progression and prevent complications [10]. If CKD progresses, it can lead to significant complications that include kidney failure (in around 2% of those with CKD) and its associated complications, such as anaemia and bone disease [11]. People with CKD are also at increased overall risk [12] of cardiovascular disease [12]- the leading cause of mortality in people with CKD [13].

CKD presents significant challenges for individuals, and these challenges are exacerbated by healthcare inequalities. Marginalised groups- including low-income individuals, ethnic minorities, and rural communities- face barriers to timely diagnosis and treatment [14–16]. Even in high income countries such as the UK, deprivation is lined to faster progression and poorer outcomes [17]. Poorer care and outcomes are associated with area-level deprivation and are greatest in those individuals with early CKD [18]. This group are known to often present with problems related to their kidney disease to urgent/ emergency care, and miss more routine monitoring appointments and testing [17]. As the prevalence of CKD increases, especially amongst an aging population and for those people with co-existing chronic health conditions such as hypertension and diabetes mellitus [19], understanding what management strategies are required is crucial. These strategies are essential to reduce the impact of CKD on people living with the condition, their social network members, and healthcare systems.

Effective self-management - the role individuals take in managing their health condition through everyday life adaptation related to behaviours, relationships, and lifestyle - has become essential for both individuals and the healthcare system. Self-management programmes for people with CKD have been shown to improve quality of life, reduce healthcare burden, and slow disease progression [20, 21]. Self-management frameworks exist in several developed countries [22], and in the UK, within the NHS Long Term Plan [23], self-management is recognised as key to delivering care.

There is a growing recognition that self-management involves different types of ‘work’ (practical, emotional, relational) in addition to ‘illness work’ (e.g. understanding and managing symptoms, taking medications) and that illness management is not an individual but a collective process. Specifically, members of people’s social networks – the constellation of formal and informal relationships (e.g. friends, colleagues, family members, peers with common interests, healthcare professionals) around a person – play an important role in enabling or preventing self-management [22]. Social networks shape self-management through the support and resources they provide (or restrict access to), by shaping perspectives towards health-related practices such as diet and exercise [23], and as an integral part of people’s identities, obligations, and valued roles (e.g., as parents, employees, friends, professionals).

There is evidence that social networks play an important role in providing self-management support, for example by reducing readmissions to hospital after exacerbations in both acute illnesses and chronic conditions such as diabetes and kidney disease [24–32]. Evidence shows that people with CKD who actively self-manage experience improved outcomes, including slower disease progression and better quality of life [26–30].

In the context of kidney failure, social support by peers has been found to influence treatment adherence and improve the overall well-being of patients receiving kidney replacement therapy [32]. Whilst there is a wealth of evidence supporting people living with significant and severe CKD, there is limited evidence around the self-management support for those with mild to moderate CKD, who are mainly cared for in primary and community care. Additionally, there is limited understanding as to how social networks work as a whole system (rather than the contributions of specific types of relationships, e.g., peers, family members) in providing or limiting different types of support.

Understanding the experiences of people living with CKD and how their illness is managed in everyday life, alongside the role of their social networks, will help improve understanding of self-management practices and inform the development and implementation of self-management support interventions. Alongside this, it is important to understand the shape and structure of both formal and informal support to understand how this impacts and influences behaviours [33]. Social network members are perceived as a crucial part of living with CKD, often being considered as a disease that is not just lived by the person with it, but rather the whole family [34].

This narrative review aims to address this gap by examining the work (emotional, practical, relational) carried out by the social network members of people living with CKD, and the role of social network support in shaping self-management behaviours and practices in different everyday settings.

## Methods

This paper presents a narrative review of the literature on the role of social networks in supporting people living with CKD. The Preferred Reporting Items for Systematic Reviews and Meta-Analysis (PRISMA) guidance [35] was used to systematically search and identify relevant literature.

### Eligibility criteria

This review included studies investigating the role of the social network and its impact on self-management for people living with CKD. Social networks were defined as ‘the structure around people that is made up of individuals or organisations associated with one or more types of interdependence (friends, family, those with common interests, work, knowledge) which together form a ‘network’ around the person’ [36–40].

The primary concept of interest in this review is the experience of people living with kidney disease, their self-management, and the role of their social network support in managing their illness. The review considered studies that provide information about social networks, self-management, and the experience of people living with CKD. A full list of inclusion/ exclusion criteria can be found in Table 1.

**Table 1:** Inclusion/ exclusion criteria

Inclusion	Exclusion
- Primary Research - Involving adults aged 18 and over - Peer reviewed	- Focus on end-of-life care - Related to pregnancy - Only including dialysis or transplant patients - Intervention-based (e.g., drug studies, dietary intake, etc.)

## Search strategy

Searches were conducted between 31st January 2024 and 7th February 2024 then rerun on 12th December 2024, and 17th July 2025 to ensure no additional articles were missed. Electronic databases: CINAHL, MEDLINE, EMBASE, PsychINFO, SocINDEX, Web of Science; Cochrane reviews; grey literature and study registries. Medical subject headings have been adapted for each database with no time constraints. Citation searching was carried out during the same periods (Table 2).

**Table 2:** Search terminology

Spider	Terminology	Search terms used
Sample	CKD Renal patients	• chronic kidney disease or chronic renal failure or ckd or esrd or renal insufficiency or kidney failure
Phenomenon of Interest	Social Network Support Carer	• AB Carer* OR caregiver* IR famil* OR friend* OR relative OR informal carer* OR “support network” OR “social support” OR social circle OR social relations OR personal communit* OR collective efficacy OR husband OR wife OR partner OR spouse OR significant other
Design	Interviews Focus groups	• Qualitative OR ethnography OR interview OR focus group
Evaluation	Experience of patients and carers	No additional terms added as covered in Phenomenon of interest.
Research Type	Qualitative Mixed methods	No additional terms added as covered in Design.

We identified published primary studies, text and opinion papers, and grey literature dedicated to the topic of self-management and social network support for patients after AKI or with CKD. Searches were conducted electronically and manually; the latter was conducted by searching for relevant articles in the reference lists of the selected articles.

A 3-step search strategy was used in this review. An initial limited search of MEDLINE (Ovid), Embase, PsycINFO, and AMED was undertaken to identify articles on the topic, followed by an analysis of the text words contained in the titles and abstracts of retrieved papers and of the keywords used to describe the articles.

The research team, consisting of lead author/researcher (BB) and research team members (IV and KV), discussed and developed the most appropriate keywords and synonyms for search activities utilising feedback from an academic librarian. Boolean operators (OR, AND), including adjacencies and truncations, were used to combine appropriate keywords and related terms.

A second search across all included relevant databases using all keywords and index terms was performed. Following an iterative process, we became more familiar with the evidence base; thus, additional keywords, sources, and search terms found to be useful were incorporated into the search strategy as appropriate. The search used keywords and Medical Subject Headings (MeSH) terms, as presented in Table 1. Search strategies were created for each database using relevant indices and free-text terms.

Articles published from January 2009 to the present were included to ensure that the included reports are relevant to the current clinical practice and legislation. The year 2009 was chosen to ensure findings would be transferable to current care. Initial searches were limited to English; however, this limitation was removed for the 2025 search to ensure all literature was found.

The third step was to include the search for additional studies by appraising and screening the reference lists of identified reports and articles, which was carried out by the primary author (BB). The titles and abstracts of all identified studies potentially eligible for inclusion in the review were screened, and full-text versions of the included articles were obtained.

### Data extraction

Following the search, all selected studies were imported into EndNote. Duplicate papers and publications that do not directly relate to the research question were eliminated. The author (BB) reviewed all potential articles, with final articles for screening decided with 2 co-authors (IV, KV) through independent article review and discussion.

The 2 co-authors (IV, KV) pilot tested the screening of titles and abstracts for assessment against the inclusion criteria for the review. All potentially relevant full-text articles were retrieved and screened for inclusion in the final review and imported into the Rayyan system. Conflicts were discussed and resolved by BB, KV, and IV. The full text of selected papers was assessed in detail against the inclusion criteria by 3 independent reviewers. 14 publications were identified as relevant through the screening process.

Standard extraction forms that included study type, participant recruitment, and type, and key themes were created in Excel and used to extract data from all eligible papers by BB. This was reviewed and amended following discussions with IV and KV. The aim was to ensure that all data relevant to addressing the research question was included. These can be found in Table 3.

**Table 3:** Study characteristics

Article	Participants recruited	PLWCKD or CG or SNM	CKD stage & where recruited from	Type of qualitative research	Location
Through the Lens of Chronic Kidney Disease: A Qualitative Study of the Experiences of Young Women Living With CKD Beanlands, Mccay et al. 2020 [43]	*n* = 11 Women 19–38 (median age 34) 55% born in Canada 91% of people included employed at time of study. ‘Most’ educated beyond high school.	PLWCKD 82% married or in common law relationship. 5 living with spouse and children.	1–3 Under nephrologist	Focus groups	Urban Ontario, Canada
“Systems seem to get in the way”: a qualitative study exploring experiences of accessing and receiving support among informal caregivers of people living with chronic kidney disease Coumoundouros, Farrand et al. 2024 [46]	*n* = 13 Women 30–69 (mean age 52) 11 White British 2 South Asian. 7 caregivers had a batchelors degree or higher. 7 were in employment.	CG (8 spouse/ partner as PLWCKD)	6 PLWCKD no on dialysis 3 PLWCKD on dialysis 3 PLWCKD post-transplant From social media	Semi-structured interviews	UK (12 England, 1 Wales)
‘It’s the empathy’-defining a role for peer support among people living with chronic kidney disease: a qualitative study Elliott, Love et al. 2022 [45]	*n* = 51 31 PLWCKD (20 men/ 12 women) 15 CG (12 spouse/ partner) (1 man/ 14 women) 6 peer mentors All patients and caregivers educated at high school level or above. Majority (45% patients/ 53% caregivers) educated to college/ university level. Most patients (74%) employed with most caregivers (53%) retired.	PLWCKD CG SNM	13 CKD stage 5 (non-dialysis) 11 CKD stage 4 CKD clinics	Focus groups (*n* = 39) Semi structured interviews (*n* = 7) 6 peer mentor interviews	Southern Alberta, Canada
Young adults with chronic kidney disease: An exploration of their relationships and support networks Coyne, Langham et al. 2019 [53]	*n* = 14 18–26 years (mean age 22) 8 men. No other information described.	PLWCKD	CKD 3–5 CKD clinic	Semi-structured interviews.	2 UK hospitals
The Perspectives of Patients on Health-Care for Co-Morbid Diabetes and Chronic Kidney Disease: A Qualitative Study Lo, Ilic et al. 2016 [49]	*n* = 66 PLWCKD = 58 Age 48–84 (median 57) 41% female CG = 8 Age 48–77 (median 66) 5 men, 3 women Only participants in semi-structured interviews described. Only ethnicity described. 5 Caucasian, 2 Asian, 1 south Asian.	PLWCKD CG	CKD 3–5 39% non-dialysis 19% HD 13% HHD 6% PD CKD clinic	Focus groups Semi-structured interviews	4 tertiary hospitals from 2 large Australian cities.
Patients’ and kidney care team’s perspectives of treatment burden and capacity in older people with chronic kidney disease: a qualitative study Hounkpatin, Leydon et al. 2020 [47]	*n* = 39 PLWCKD = 29 55% men, 45% women Aged 60–90 97% white 73% married or living with partner Clinicians = 10 90% female, 20% consultant nephrologist 40% renal nurse specialist 20% renal dieticians 10% renal pharmacist 48% secondary school education 27% post secondary school training 25% degree. - Education not separated between pt and clinicians.	PLWCKD Clinicians	CKD 3–5 Pre dialysis 100% 38% CKD 3 41% CKD 4 21% CKD 5 2 GP surgeries (11 PLWCKD), 2 CKD clinics (18 PLWCKD)	Semi-structured interviews (PLWCKD) Focus groups (Clinicians)	UK
Treatment Decision Making for Older Kidney Patients during COVID-19 Porteny, Gonzales et al. 2022. [50]	*n* = 76 39 PLWCKD (56% non white) (70–90+) (59% women) 17 CG (40–89) (71% women) 20 Clinicians (85% nephrologists) Patient- 56% white 33% black Caregivers 76% white. 24% black. All patients/ caregivers educated to highschool level. With 28% patients and 35% caregivers educated to post graduate level.	PLWCKD CG Clinicians	CKD 4–5 7 on dialysis	Semi-structured interviews	Boston, Portland, San Diego, Chicago. USA
Spirituality, Coping, and Resilience Among Rural Residents Living with Chronic Kidney Disease Pham, Beastley et al. 2020 [44]	*n* = 80 PLWCKD- 45 CG 15 Religious leader 4 16 people in focus group- unclear of make up 95% high school educated.	PLWCKD CG Religious leader	CKD- all stages – Not specified	Semi-structured focus groups (64) in-depth interviews (16)	Robeson County, North Carolina, US
“Maybe They Don’t Even Know That I Exist”: Challenges Faced by Family Members and Friends of Patients with Advanced Kidney Disease O’Hare, Szarka et al. 2017 [56]	*n* = 17 23% spouses 65% other relatives 12% friends 77% women Age 42–81 Mean age 60 35% black, 56% white, 11% other All PLWCKD veterans	CG SNM	CKD 44% not on dialysis 37% HD 19% PD	Semi-structured interviews	Puget sound, Washington, USA
Cognitive impairment in patients with chronic kidney disease-Next of kin’s experiences Schjerlund, Agnholt et al. 2021 [52]	*n* = 10 50% female All spouse/ partner 41–77 (mean 66) 70% employed. 20% home dwelling children.	CG	CKD 5 with cognitive impairment 2 Pre dialysis 1 HD 2 HHD 3 PD 2 transplant CKD clinic	Semi-structured interviews	Denmark
Using an international online forum to explore perspectives of caregivers of patients with chronic kidney disease Tuckey, Duncanson et al. 2022 [51]	*n* = 112 creating 159 posts 48% husband/ male partner 4% wife/ female partner 34% parent	CG	Online internet forum	Qualitative content data analysis	Unclear as could be anywhere
Peer support for CKD patients and carers: Overcoming barriers and facilitating access Taylor, Gutteridge and Willis. 2016 [57]	*n* = 26 15 PLWCKD (8 women, 7 men) (36–77 years old) 11 CG (8 women, 3 men) (52–67 years old) Caregivers (11) Rural (6) Urban (5) Working (4) Education to GCSE or above (7) Patients (26) Rural (9) Urban (17) Working (3). Education to GCSE level or above (9).	PLWCKD CG	CKD 5 3 pre dialysis 2 training for HHD 2 awaiting transplant 11 HHD 4 HD 2 PD 1 transplant CKD clinic	Interviews	6 NHS hospital trusts Rural (15) Urban (11)
The self-management experience of patients with type 2 diabetes and chronic kidney disease: A qualitative study Shirazian, Crnosija et al. 2016 [76]	*n* = 23 14 men, 9 women. Age 18–79 (64 mean) 18 participants married 17 white, 5 black, 1 Native-American. 8 had graduate degree.	PLWCKD	CKD 2–5 & T2DM CKD clinic	Semi-structured focus groups	New York, USA
Supporting People with CKD to Self-Manage Lightfoot, Wilkinson et al. 2025	*n* = 22 11 men Age 48–88 years (mean 71) CKD stage 1–5 20 participants white British 1 Indian 1 Pakistani 7 university level education 4 college level education 6 high school level education	PLWCKD	Recruited from outpatient clinics	Semi-structured interviews	Leicestershire

### Quality appraisal

All studies included were qualitative and were assessed for their quality utilising the CASP: Qualitative Studies checklist [41]. Each full-text paper was quality assessed by BB in parallel with data extraction. This quality assessment was discussed with IV and KV, and any conflicts resolved with discussion.

### Data synthesis

A narrative synthesis was undertaken following the stages outlined in the Guidance on the Conduct of Narrative Synthesis in Systematic Reviews [42]. Data was collected that addressed the key outcomes for this study, with textual data collated on data extraction spreadsheets. This focused on social network involvement in care for people with CKD stages 1–4 across the lifespan. Data was collected using aspects of thematic synthesis, with a thematic framework being developed. Themes were developed and refined as necessary with the addition of each study. Following the initial author (BB) development of themes, these were discussed and further refined with IV and KV. This was done through discussion and repeated review of the initial studies, with any conflicts resolved through discussion.

## Results

The 14 included papers used semi-structured interviews [7], focus groups [2], both semi-structured interviews and focus groups [4], and 1 qualitative content data analysis of an online peer support group. None of the studies included within the review had a primary focus on social network involvement in health.

A total of 560 participants were included in the 14 studies, with 287 people living with CKD, 218 social network members, 30 clinicians, 6 peer group mentors, and 4 religious leaders. 10 of the 14 studies only included people living with CKD stages 3–5. This means social network implications for early CKD are not well described by these studies. There was limited description of socio-economic backgrounds, with educational level (9/14 papers) and employment (5/14 papers) being described.

People aged 18–90 were included, with people living with CKD, age ranges, gender, and ethnicity being well described. For social network members, age and ethnicity were less well described. Only 157 of the social network members had relationships described, of these the majority, 150 (96%) were family members − 95 marital or non-marital spouses, 39 parents, 10 siblings, 6 children – and only 4 were friends and 3 were another relation. This lack of information and the almost exclusive focus on support from family members may therefore mean that these studies may not well describe the impact of the wider social network on care.

The studies were carried out in the UK (5), United States of America (4), Canada (2), Denmark (1) and Australia (1), with few of the studies reporting whether the patients were from rural or urban backgrounds. 11 of the 14 studies recruited participants only from nephrology outpatient clinics, with 1 study recruiting from both GP surgeries and nephrology outpatient clinics, 1 study recruiting through social media and 1 study using public posts in an online forum. A table for the papers can be found in summary Table 1.

### Thematic development

Data from all included studies were grouped based on evidence regarding social network members and people living with kidney disease. Through interpretive synthesis of the included studies, we identified four recurring themes that illustrate the complexity of social network involvement in CKD self-management.

4 themes were developed:

- The burden of kidney disease uncertainty.
- Everyday challenges of managing CKD.
- ‘The loneliest disease’.
- The role of Peer Support.

#### Theme 1. The burden of kidney disease uncertainty

Many people living with kidney disease were uncertain about what CKD is and what it means for their health. Thus, some people overestimated the impact of their diagnosis, “*35% kidney function - I thought that was a death sentence*” [43], “*I just lost it*,* I just fell apart”* [44]. Whilst others reported that they did not appreciate the impact of the severity of their illness- “*the doctor said ‘Or we can wait until you need a kidney transplant’*,* and that’s when it really hit me*” [43]. These experiences were in relation to the uncertain trajectory of illness progression and diagnostic and communication hesitancy on the part of healthcare professionals.

Participants reported that they found information from kidney specialist teams to be beneficial, but due to the limited access to kidney specialists found they had persistent knowledge gaps that were unable to be addressed by other non-kidney specialists [45]. Both people living with CKD and social network members found that meaningful information helped empower and engage them, and removed fear, and that this was best sourced from kidney specialists [43]. Meaningful information was deemed to be clear, accurate, and directly related to CKD [43], tailored to the context of the patient/caregivers’ situation and given empathetically [46]. In some studies participants reported that outside the world of kidney specialists they experienced healthcare professionals with limited understanding of kidney disease. They felt non- renal professionals were unable to provide them with the education and information they required [46] or offered advice that conflicted with that given by kidney specialists [47]. In the Beadlands study [43], it was found that nephrology input was required to be able to ensure information was given at a time when it would be beneficial and appropriate. This need for nephrology specific input led to further challenges for people with CKD due to the uncertainty and unknowns they were facing as to what they can expect and what they may need to do: “*you can’t always live in the anxiety of the future*” (young woman with CKD) [43].

### Uncertainty in illness progression

The progression of CKD is described by social network members as an initial silent trajectory, without specific recognised signs, with many studies reporting that patients and caregivers were not aware of the impact and chronicity of CKD at the point of diagnosis [45]. The absence of observable and experiential signs of CKD has a serious impact on the mental health and anxiety levels of people living with CKD and the social network members who support them [48]. The experience of diagnostic uncertainty felt like a “*black cloud*” hanging over them, whilst waiting for kidney function to deteriorate (social network member [45]. In one study participants reported that, as a way of reducing uncertainty, they wanted to change their living arrangements and ensure they lived near a renal unit in the future, in preparation for their illness deteriorating [47].

### Diagnostic and communication hesitancy by healthcare professionals

People with CKD and their network members felt unsupported and misunderstood by healthcare professionals [43, 49]. Advanced care planning, involving prognostic discussions, was viewed by healthcare professionals as an important part of shared decision making [50]. However, the perceived challenges associated with these discussions led to diagnostic and communication hesitancy. Kidney specialists felt unsure about what information to share, and how to strike, what they saw as, a balance between overloading/ frightening people and providing them with enough information to be able to actively participate in disease modification [47]. In contrast, participants reported that information on CKD as an illness given to them by a healthcare professional was important to help them understand their condition, plan for the future, and manage illness uncertainty [51]. Additionally, when they received oral information from kidney specialists, this assisted them in contextualising the information and reduced their burden “*I believe that the oral (information) is better coped with than written. You must get home and prepare yourself for a read. That is not easy when you are with someone who is sick*” [52].

People living with CKD reported a lack of knowledge and understanding around CKD management from non-renal professionals, which had an impact the quantity and quality of the information provided, contributing to the uncertainty of illness. Diagnostic and communication hesitancy could also be due to a perceived attempt by healthcare professionals to protect people from excessive worry, or a lack of knowledge (by non-renal professionals), but had the same impact on people living with CKD and social network members, leading to experiences of uncertainty and anxiety [43, 46, 53].

Additionally, when information was not provided, or poorly provided [45] this directly impacted people’s choices about treatment [47] and led to an inability to fully prepare for the future [46]. For example, some patients perceived their CKD not to be a significant problem as the health professionals providing their care displayed a lack of concern, with one patient reflecting “*I wasn’t in pain*,* so I didn’t think anything was wrong with my kidneys*” [53]. Both people living with CKD and social network members reported a reliance on the information and advice that healthcare professionals provided, and that this helped them self-manage successfully, and reduced uncertainty [43].

#### Theme 2: everyday challenges of managing CKD

Many of the included studies highlighted the daily challenges that people living with CKD and their network members face [43, 45, 47, 54]. These include: managing fluctuations in health-energy levels [43], body image [43] and multiple competing medical conditions [47]. The psychological challenges experienced [49, 51, 55] included anxiety, depression, and negative emotions.

Managing multiple medical conditions was challenging due to the need to manage the medications burden, alongside the substantial work required in co-ordinating formal and informal aspects of healthcare [47]. This included arranging transport for all the different appointments, organising prescriptions, managing appointments and requirements that were often spread between primary and secondary care, and sometimes between several different secondary care organisations. Patients found this overwhelming and sometimes exhausting “*I just get a bit fed up sometimes of all these visits* [47]”.

Participants also reported emotional and psychological challenges when accessing information and tangible support [45]. This was related to the complexities of initiating, managing, and maintaining relationships [53] and managing the expectations of others. For close social network members, this was sometimes about managing roles and responsibilities “(my) *husband gets tired of me not being able to do stuff*” [43]. Whilst when dealing with non-kidney specialists, this was sometimes about managing their expectations and limited knowledge of CKD in relation to other healthcare needs- for example during and after pregnancy- “*the pressure to breastfeed was unbelievable*” [43]. Frustration and depression were often triggered by the everyday challenges faced, but when they managed to successfully overcome challenges, this gave participants hope for the future [43]. Young women reported that when challenges were mitigated or addressed, they were better able to focus their attention on fulfilling their everyday roles within life, have life ambitions, and, in general, get on with their lives.

Where people adopted health promoting behaviours they did not necessarily associate these with self-management and kidney health. One participant shared “I was drinking more water anyway, but I didn’t know that this was helping my kidneys” [53].

Social network members reported daily struggles with their own emotions, the impact on their lifestyle, and supporting others both emotionally and practically [51]. Social network members reported that when their loved ones experienced fluctuations in health, different levels and types of support were required from them, and this often led to caregivers putting their own physical and mental health needs last [55]. This sometimes led to negative emotions and a negative impact on their quality of life [51].

Social network members reported that they are often expected to work within the inflexible, unfamiliar, and disjointed health and social care systems [56], while also feeling poorly integrated into them. They felt that they were “*pushed from pillar to post*” when looking for support [46] within the healthcare system. The complex structure of healthcare systems placed a distance between social network members and healthcare professionals, and this made healthcare professionals difficult to access [46]. This experience of challenging interactions within the healthcare system was reported by people living with CKD and caregivers, and there was no discernible difference when people had wider support from their own social network.

Social network members felt they were often expected to fill gaps in the healthcare system, without being integrated into it or acknowledged. This made them feel invisible and taken for granted by healthcare professionals [56], which caused frustration and negatively impacted their views about the healthcare system and their relationships with healthcare providers [46]. Whilst social network members recognised that the healthcare system was there to support the “*patient*” as caregivers, they felt that they “*didn’t count*”, were “*dismissed more than supported”* [46], had a sense that, as far as healthcare providers were concerned, “maybe they don’t even know I exist” [56].

#### Theme 3: ‘The loneliest disease’

People living with CKD and their social network members frequently reported feeling lonely and isolated [43, 45, 46, 53]. This was partly attributed to a lack of societal awareness and visibility of CKD, which had an impact on relationships with employers, friends, and partners. One patient shared “*I’ve never met anyone else with this. It makes you feel like you’re the only one*” Participants expressed frustration at having to constantly explain and justify their illness to others, often feeling that CKD lacked recognition compared to other conditions “*People aren’t wearing ribbons or bands. It’s not something that people intrinsically understand*” [43]. This led to CKD being described as ‘*the loneliest disease’* [45].

The invisibility of illness, combined with the daily demands of self-management, amplified feelings of isolation. To avoid anticipated stigma, many individuals chose to withhold or minimise disclosure of their conditions in both social and work settings. Young adults reported difficulties disclosing their illness in romantic and friendship contexts often led to rejection: “*as soon as I mention*,* like me being poorly and dialysis and the fact of a transplant they are gone*” [53]. This further exacerbated their sense of isolation.

### The strain on work, valued social roles, and daily life

Living with CKD placed a significant strain on daily life, especially in managing work responsibilities. Many individuals struggled to balance their health with professional demands, with some reporting feelings of uselessness due to their inability to work: “*laying there*,* feeling bad about everything….I can’t work anymore”* [44]. Employers and colleagues were felt to frequently underappreciate the implications of the illness, contributing to misunderstandings and limited accommodations [43].

This burden extended to social network members, who often took on emotional and practical caregiving roles. The time required to do this led to concerns over how this could be managed, as it was reported to significantly conflict with their work commitments [47, 51, 52]. The emotional labour of caregiving often went unrecognised, increasing feelings of stress and caregiver fatigue.

### Shifting relationships and coping with emotional burdens

As CKD progressed, both patients and their social network members experienced a reduction in the size and strength of their social network [56]. This was attributed to lifestyle changes that limited access to friends and family [55], reduced participation in social activities [47], and self-imposed distancing from others as a way of reducing the burden on them [45, 46]. Social network members also noted that caregiving responsibilities increased their isolation from their own friends and family, who participants felt did not understand or support their caregiving role [46].

The emotional and physical toll of caregiving was reported as significant, with many struggling to balance these responsibilities alongside personal needs. This led to missed social activities, difficulty in planning, and feelings of self-neglect [46, 52]. Some caregivers reported feeling unsupported by friends, family, clinicians, while they prioritised the needs of the person living with CKD, they neglected their own needs [55]. These dynamics could lead to feelings of resentment and anger towards people living with CKD [51] and negatively affected relationships, including spousal separation [50]. People living with CKD and their caregivers employed coping strategies such as humour and maintaining a positive outlook to manage the emotional weight of their experiences [47, 51].

#### Theme 4: the role of peer support

Peers- people with CKD diagnosis or their network members were found to offer shared experiences and emotional validation, helping both patients and caregivers navigate the uncertainties of CKD, One patient shared “*I’ve never met anyone else with this. It makes you feel like you’re the only one*” [53] Access to peer support helped maintain existing connection and build new ones while living with CKD [45], and was most beneficial when there was good rapport and where support could flex with the requirements and wellness of the person who was being supported [55].

Peer support was deemed valuable by people living with CKD and social network members alike. Peers with kidney disease were seen as a resource in navigating uncertainty by helping to contextualise the future course of CKD progression, which allowed them to feel in control [55].

The support of people with a similar lived experience was important, as it was felt that their existing social network or healthcare professionals [45] could not fully relate to their situation “*no-one can understand this particular hell we are both in*”. This was because participants felt peers would be more honest about the challenges faced when living with CKD. They were able to be honest about their own illness without evoking unwanted sympathy or pity, which they experienced from their other social network members. The support received from peers was highly valued “*I don’t want sympathy… it’s the empathy*” this alleviates feelings of isolation [45].

How peer support, deemed to be valuable, was sourced varied between studies. Respondents discussed two broad types of peer support: formal and informal. Formal peer support included connections through healthcare professionals, e.g., being invited to attend a peer support meeting or being linked to a peer support mentor [45, 54, 55]. Informal peer support was where people found others with similar experiences through chance encounters, e.g., knowing others with CKD, meeting them in waiting rooms, or online. Participants in one study reported that they did not build meaningful peer connections in some of the informal encounters they had, such as meeting other people with CKD in waiting rooms [45]. Such incidental encounters did not meet al.l their needs [55], such as answering questions about disease progression or treatment options, and there was a requirement for a more formalised structure to peer support. However, other participants found that the type of formalised peer support that was available to them (such as information sessions from people living with CKD) had a structure restrictive to developing relationships and was less valued [55] than informal peer support.

Organised, formal peer support was mostly discussed in negative terms. This was because respondents felt such support may be unavailable when needed, and if available, may require commitment that may be difficult to make [55]. Additionally, such an environment was seen as uncomfortable for people who are shy, not sufficiently confident, or not very sociable [55]. It was also recognised that for formalised peer support to be beneficial, it was important that people were able to maintain ‘normal’ friendships outside such groups. These were highly valued relationships that did not focus on illness [53]“*I think they gave me the chance to meet other young adults on PD [peritoneal dialysis] but I did not really want to meet someone anyway. I just wanted to get on with my life and see my normal friends*” [53]. Within the included articles, there was a lack of discussion around socio-economic variation and whether any additional factors would be required.

Both Social network members and people with CKD utilised online forums to find peer support to share intimate experiences and feelings, which they might have felt uncomfortable doing in a face-to-face formal (or informal) peer support setting [43, 51]. They found it beneficial to connect with peers in an online forum as these offered answers to very specific questions, alleviated feelings of isolation, and provided necessary social support, such as being able to share their emotions and frustrations anonymously and without feeling judged [51].

Another type of non-illness-focused support that was valued was spiritual peer support. This was reported by one study [44], which found that people living with CKD experienced a supportive community within religious groups. Hospitals and clinics were not considered to be gathering places where one can experience community building and support, which was in contrast to their experiences in church. They also found that spirituality appeared to give a means of understanding and contextualising CKD to their everyday life, which led to a more positive experience. This, in turn, led to improved self-management, including more productive interactions with health care professionals. Participants reported that even when their health prevented them from attending church, the churchgoers came to them, meaning that the support provided by those within the church group continued as illness progressed. This suggests that a range of community resources might be important for the emotional wellbeing [44] of people with CKD and members of their support networks.

## Discussion

This is the first narrative review that explores the role of social networks in supporting the self-management of people living with CKD. Although most included studies originally focused on spouses and immediate caregivers, our synthesis shows that a wider array of relationships- including distant family members, peers, friends, healthcare professionals, religious leaders and online communities-, provide practical, emotional, and informational support and shape the daily experiences, self-management practices, and health trajectories of people with CKD. Experiences specific to CKD, such as diagnostic ambiguity, diagnostic and communication hesitancy, and the social invisibility of the illness, influence how and when network support it mobilised.

*Understanding uncertainty in CKD and the role of social networks in CKD self-management* Chronic disease literature demonstrates that self-management is not solely an individual responsibility but is a relational practice deeply embedded in social contexts and involving negotiations of valued identities and social roles [57]. In contrast to other long-term conditions, CKD presents unique self-management challenges due to its frequently silent progression and diagnostic ambiguity, particularly in the early stages. For both individuals with CKD and their network members, the uncertainty about “what is the illness” and the lack of clarity as to “what needs to be done” also makes it difficult to engage network support and distribute responsibilities, as the work is not specified. This ambiguity leaves it to individuals with CKD and their network members to work out the meaning, priorities, and specifics of everyday self-management. This is done ad hoc, leading to disjointed efforts of arranging care, poor engagement of support networks, a sense of anxiety and isolation, and with possible negative impact on adherence to treatment and lifestyle modifications [58, 59].

### Limitations of the traditional medical model in CKD care and the burden this creates

While professional support interventions are widely recognised as essential for self-management [60, 61], this study highlights a mismatch between CKD’s uncertain early trajectory (i.e., non-linear illness progression) [62] and the healthcare system’s reliance on a traditional medical model [63]. This model often fails to recognise CKD as an actionable diagnosis until later stages, meaning that support only becomes structured and consistent once individuals reach late-stage kidney disease. This reinforces a care gap in earlier stages- diagnosis without treatment pathways- which leads to diagnostic and communication hesitancy on the part of healthcare professionals and undermines both formal and informal self-management support. While translation of medical knowledge into everyday practices is always complex [64, 65], in CKD there are also tensions as generalists often provide conflicting or incomplete information. Renal specialists were valued for contextualising information, but limited access left patients navigating contradictions and uncertainty [66, 67]Whilst other models have been designed and implemented in the care of patients with CKD [66, 68], many participants in the included studies reported that meaningful engagement with the healthcare system only became consistent when they approached kidney failure. This makes self-management at earlier stages more precarious and shifts the burden onto people with CKD and members of their informal networks. People with CKD and network members often had to independently source and interpret information [69]. A key implication for service design is the need for accessible, stage-appropriate input from appropriately trained specialists to prevent knowledge gaps that inhibit effective self-management.

### The invisibility of CKD and attrition of network support

Whilst many other medical conditions have no visible outward signs, such as diabetes or hypertension, this review highlighted that the invisibility of CKD is exacerbated by its societal invisibility. Unlike cancer or cardiovascular disease, which often elicit broad public support and awareness, CKD remains poorly understood, and participants within the studies often reported that the absence of a collective understanding of society further impacted their lived experience of disease management. Similar findings in conservatively managed kidney failure patients describe CKD as “intangible” with symptoms often mistake for ageing, leading to uncertainty and loss of control [70].

Social network members of people with CKD often fail to understand why care is needed, what role they should play, or what outcomes to expect, leading to stress, guilt, and isolation. Limited recognition within healthcare adds to caregiver burden and poor quality of life [71, 72]. This aligns with previous research indicating that healthcare systems prioritise end-stage CKD care, leaving those with earlier stages and their carers feeling invisible [73].

The lack of visible symptoms, coupled with poor public and clinical recognition of the early-stage CKD, creates significant barriers to mobilising social support and leads to the erosion of available informal support. This is particularly problematic over time when people with CKD need more help as their illness progresses to later stages. Efforts to better integrate formal and informal networks, such as community health and wellbeing workers (as found in the UK) [74] could offer pathways forward. These roles may bridge gaps by ensuring early identification of needs, coordinating community resources, and facilitating ongoing support for both the patient and social network members.

### Peer support as informal translation and belonging

Peer support is widely recognised as an important aspect of self-management support. In early CKD, where formal guidance is limited, peer support helps individuals understand illness progression and contextualise it with everyday life Informal connections and online peer support provided flexible access to discuss their illness openly and without fear of pity or judgment. Religious and community groups helped address key aspects of everyday self-management: opened spaces to make sense of illness experiences, identify meaningful social roles, and offered a sense of belonging especially illness restricted social activities. The flexibility and accessibility of peer support were central to its effectiveness, suggesting that rigidly structured programs may not meet the diverse needs of CKD patients and caregivers.

While peer support exists in many chronic conditions, its function in CKD as a “translational bridge” between uncertainty and action appears especially vital, given the lack of structured support currently available in early stages.

### Limitations

Most included studies focused on individuals with later-stage CKD who were already under nephrology care. As a result, the experiences and support needs of those with earlier-stage CKD, who are often managed in primary care, are underrepresented. This reflects a broader trend in healthcare overlooking people with early CKD.

The findings from this study draw on qualitative synthesis, which, while rich in depth, may limit generalisability. The experiences of CKD patients and their social networks can vary widely depending on cultural, economic, and healthcare contexts.

This divergence between research design and actual findings signals the importance of expanding the conceptualisation of social network support in CKD that goes beyond the household.

## Conclusion

This review reveals how CKD is shaped by diagnostic and communication hesitancy, delayed and inconsistent formal support, and social invisibility, especially in early stages. Our findings indicate that in the absence of a clear care pathway, the current structure of support is only likely to work well for people with easy access to a specialist and informal peers. Such support can enable understanding of illness progression, meaningful engagement with self-management practices, and open up access to sustainable support from wider networks, while also reducing uncertainty and ambiguity. However, access to such support is only likely to be available to a small number of people with CKD. This demands a new approach to providing and integrating informal and professional care. Three key insights have arisen from this study: 1. Early-stage CKD requires clearer communication at the point of diagnosis, and proactive support structures- both formal and informal- to prevent isolation, ambiguity, and delay in care. Healthcare systems need to consider how this can be embedded into care.

1. Early-stage CKD requires clearer communication at the point of diagnosis, and proactive support structures- both formal and informal- to prevent isolation, ambiguity, and delay in care. Healthcare systems need to consider how this can be embedded into care.
2. Peer support and a sense of community belonging play a vital role in helping individuals navigate uncertainty and reclaim agency. Recognition of the importance of peer support and ensuring patients are appropriately signposted to these groups will assist in reducing isolation.
3. Service design needs to consider how to embed renal specialist input, ensuring accessibility across the CKD trajectory and supporting the development of knowledge to reduce anxiety. This could include training non-renal staff to offer accurate advice, and utilising community facing roles, such as social prescribers, to bridge the gap between clinical and daily life.

Future research should investigate how early-stage CKD patients and their network members can be better supported through integrated models that combine specialist knowledge, peer support, and community resources. A broader and more inclusive understanding of the role of social network members and the work that is undertaken by them is essential for improving CKD care.

## Data Availability

The data generated and analysed during this study are available from the corresponding author.

## Abbreviations

CKD: chronic kidney disease
GFR: glomerular filtration rate
eGFR: estimated glomerular filtration rate
AKI: acute kidney injury
UK: United Kingdom
PRISMA: Preferred Reporting Items for Systematic Reviews and Meta-Analysis
